# The Hurt of Judgment in Excessive Weight Women: A Hermeneutic Study

**DOI:** 10.5539/gjhs.v7n6p263

**Published:** 2015-04-23

**Authors:** Neda Mehrdad, Nahid Hossein Abbasi, Alireza Nikbakht Nasrabadi

**Affiliations:** 1 Endocrinology and Metabolism Research Center, Endocrinology and Metabolism Clinical Sciences Institute, Tehran University of Medical Sciences, Tehran, Iran; 2 International Campus, Tehran University of Medical Sciences, Tehran, Iran; 3 School of Nursing and Midwifery Ahvaz, Branch Islamic Azad University, Ahvaz, Iran

**Keywords:** excess weight, hermeneutic-phenomenological, lived experience, women, the hurt of judgment

## Abstract

Excess weight is one of the increasing problems of the present society and one of the threatening health conditions around the world. Despite many efforts for prevention and treatment or even surgery, the process of excess weight is not decreased in the world. While most of the studies conducted on excess weight concentrated on the issues why people get excess weight or how the prevention and treatment of excess weight must be performed, there is lake of knowledge about what excessive weight people really experience in their daily life. Understanding the lived experience of excess weight in women is linked with their health and society’s health while it indirectly develops the nursing knowledge to improve the quality and access to holistic health care in excessive weight women. The aim of study was to describe with a deeper understanding, the lived experience of excess weight in women. Using a hermeneutic phenomenological approach and a van-manen analysis methods, in depth semi- structured interviews were conducted with twelve women who had lived experience of excess weight. The hurt of Judgment was the main theme that emerged in the process of data analysis. This theme was derived from three sub-themes including social judgment, being different and being seen. These findings can prove helpful in promoting the nursing knowledge concerning a holistic approach in communicating to excessive weight people.

## 1. Introduction

Excess weight is one of the increasing problems of the present society and one of the threatening health conditions around the world. The physical, psychological, social and economic effects of excess weight made it as one of the most complex health problems in the present century, and the number of excess weight people in the world is continuing to increase (Bjorg, 2012; WHO, 2014). Based on a report by World Health Organization, the prevalence of excess weight not only has occurred in developed countries but also is continuing in developing countries (WHO, 2013). Due to the change in Iranian lifestyle and nutrition, the prevalence of excess weight has apparently increased in recent decades (Jalali-Farahani et al., 2014; Ezzati Rastegar, Peyman, Taghipour & Esmaily, 2012). The phenomenon of excess weight defined function, BMI 30 and above it, creates a wide range of chronic systemic diseases such as diabetes, heart disease, stroke, osteoporosis, gall bladder diseases, and developing varieties of cancers (endometrial and breast cancer in women) and maybe associated with other problems such as, menstrual irregularities and increased risk of surgery (WHO, 2014; CDC, 2014). Psychological disorders such as depression, anxiety, low self-esteem, feelings of discrimination and dissatisfaction associated with excess weight (Nies & Mcewen, 2011). From social perspective, the excess weight people are also vulnerable, face challenges in interacting with society during their lifetime, and worried about being repeatedly judged by others (Puhl & Hever, 2009; Matterud & Mphill, 2011). Excess weight can also negatively affect people’s capabilities for experiencing an active and dynamic life (Asnawi, Rory, Johannes, Christopher & Anna, 2011; Glinianowicz, Zygmontowcz Owczarek, & Elibol, 2014). Although there may be similarities of lived experience of excess weight in women, some excess weight women do not report such negative feelings exactly the same way (Saelens et al., 2002). The range of positive and negative feelings differs in different cultures, ethnicities and countries and is differently expressed. Excess weight is not a negative experience in the Middle Eastern, North African, and South Asian countries, and it is even considered as wealth for the individuals (WHO, 2013). From gender perspective, Excess weight bears a high significance for women. The findings of the studies also show that Excessive weight women are more concerned about their weight and appearance than Excessive weight men so that a sense of joy, charm and sociability is more reported by natural weight than excessive weight women (Depies, 2011). Despite many efforts for prevention and treatment, the process of excess weight is not decreased in the world, and the average weight is increasing which can cause more health problems (WHO, 2014). While most of the studies conducted on excess weight concentrated on the issues why people get excess weight or how the prevention and treatment of excess weight must be performed (Moyer, 2012; Fitch et al., 2013), there is lake of knowledge about what excessive weight people really experience in their daily life. Understanding of the lived experience of excess weight in women is one of the issues which must be taken into consideration. Heath care providers which emphasize the whole person, with a holistic approach can to gain a deeper understanding and awareness of lived experience of excess weight in order to offer realistic advice to women. In Iran, there is no study conducted on lived experience of excess weight in women, although there have been few studies conducted on life quality and template food (Jalali-Farahani et al., 2014; Ghorbani, Ziaee, Sadeghi & Asefzadeh, 2012).

Despite Women having similar sex, they have different experiences of living with excess weight in different social classes (WHO, 2013; Fikhan & Rothblum, 2011). Understanding women’s lived experience of excess weight is linked with their health and society’s health while it indirectly develops the nursing knowledge to improve the quality and access to holistic health care in excessive weight women. Therefore, the present study was conducted to gain an understanding about women’s lived experience of excess weight in order to gain a deep insight about life of excessive weight women.

## 2. Methods

Since the aim of the present study was to gain an understanding about women’s lived experience of excess weight, and each of them has a different perception about excess weight due to certain conditions, the qualitative phenomenological approach was used (Van-manen, 2006, 2014). Six activities of van-manen which used in the study were: 1) Turning to the nature of lived experience, 2) Investigating experience as we live it, 3) Reflecting on the essential themes, 4) Describing the phenomenon in the art of writing and rewriting, 5) Maintaining a strong and orientated relation to the phenomenon and6) Balancing the research context by considering the parts and whole.

### 2.1 Participants

Participants in the present study included 12 women above the age of 18 years (average of 43/2 years) and BMI 30 or above (average of 35). Besides, we tried to choose them from different social and cultural levels (age, education, job, marital status, ethnicity and various BMI) that entered through purposeful sampling into the study. The researcher visited excessive weight women that could well transmit their experiences. In the first meeting, the researcher talked about the aim of the study, the procedure, the role of the researcher and participants, and their interest and enthusiasm to participate in the study; the consent forms and their demographic features were filled out by participant, and the item and place of next meeting was coordinated with their agreement.

### 2.2 Data Collection

Interview, which is the most common method in qualitative studies, was used to gather data (Van-Mannen, 2006; Holloway, 2010). The interviews were semi-structured and performed individually and started with questions such as “What comes to your mind when you hear the word excess weight”?“ And “what does life with excess weight mean to you?”, but the sequence of questions was not the same for each participant because the process of the questions depended on the process of the interview and each participant’s answer (Holloway, 2010). To evolve depth interviews, follow-up questions such as “Can you call me an example?” was asked. All interviews were held in a safe place so that privacy of the individuals was observed. During the interview, the researcher tried to pay attention to all the verbal and non-verbal communication of the participants. Without guiding interviews to any specific direction, the researcher tried to enter into the lived experiences of the participants. Depending on each participant’s conditions, each interview lasted 30 to 62 minutes. A total of 15 interviews were conducted (3 participants were twice interviewed). The participants were also asked, if they wish, to present their memories of living with excess weight. Thus, two participants delivered their handwritten experiences (plus interviews).

### 2.3 Data Analysis

Data analysis and data gathering were simultaneously done. With participants’ permission, the interviews were recorded and after many times of listening, meaningful units were determined to be typed as soon as possible in order to provide feedback for adequacy and saturation of data, and it continued till the data were repeated. In the present study, overall and selective approach was used to separate the content analysis. At first, text of each interview was considered as a whole, and the major meaning of a text was described in a paragraph as a whole. In selective approach, meaningful sentences and units, which described the phenomenon on interest, were separated. Concerning similarities among sub themes and their organization as well as hesitating over themes of innate phenomenon of lived experience of excess weight, the major theme of the hurt of judgment and sub themes of social judgment, being different and being seen were achieved. In the present study, accuracy of data was determined through four criteria of Lincoln’s and Gaba’s reliability, credibility, transferability and objectivity (Burns & Grove2009). To determine acceptability of data, there was a constant challenging with the issue and data. Composing was also used in data gathering. To determine data objectivity, all the activities were recorded, and a report of research process was provided.

### 2.4 Ethical Consideration

This study was approved by the ethics committee of Tehran University of Medical Sciences and also Researchers avoided mentioning women’s ethnicities and BMI.

## 3. Findings

The major theme of “*the hurt of* *judgment* “, which emerged through the data of the present study, dealt with lived experience of excess weight and was based on the aim of present study-understanding the meaning of lived experience of excess weight in women. Women experienced profound challenges as they communicated with others. Most of the women considered “*the hurt of* *judgment*” as one of the important factors in physical and mental health. In other hands all of them mentioned the “*the hurt of* *judgment*” by social world verbally and none verbally. They found themselves in a situation in which it was worried to encounter in society. Besides the situation they felt judgment by others. They did not want to evaluated and prejudice by others because their body size. In this theme, there are three sub-themes of *social judgment*, *being different* and *being seen*.

### 3.1 Theme 1. Social Judgment

Participants’ statements represented a negative attitude towards gaining weight in the society. Most of the participants complained about social world. One of the women said, *“We judge by our eye not our reason. People are superficial observers. They judge us by our appearances,”* or *“People consider those suffering from cancer being bald because of chemotherapy far better than those who gain weight or are obese.”*

Attending ceremonies or celebrations was another important experience repeatedly mentioned by the participants. In this regard, one of the women said, *“When a table is set in a ceremony, as we sit to eat, they say how much she eats while the fat women don’t eat much as they say”*. Participants also experienced anxiety of being in a community to receive health services. One of the women said, *“My veins are difficult to find. When the nurses or anesthesiologist couldn’t find my vein for injection, they deliberately hit me and said I had been stuffed or I had eaten too much or you couldn’t eat food tonight”*. The same participant, while buying clothes, said, *“When I want to buy clothes, they say they ran out of my size. It makes me sad since I haven’t yet said for whom I want to buy clothes.”* The range of social judgment concerning women existed in other social aspects. In this regard, one of them said, *“When I decided to take my driving license, they told me that I’m fat and not authorized to take it. They asked me to perform full tests.”*

Some women found ways to deal with social judgment. One of them said, *“When I want to go jogging, I put in my ear hands free not to hear the people because it’s possible someone says something and disturbs me. I don’t like them to call me squab. It hurts me.”*

Another participant said, *“I’m silent. It’s better. It means you didn’t hear anything to answer. It means at least you didn’t hear.”*

The role of social judgment in some cases had a great impact on some participants’ regarding losing weight; that is, it was on obstacle in losing weight. One of the participants said, *“Whenever I went on diet and my classmates said I lost my weight, I gave up the diet. Now, I’m going on a diet again”*. On the other hand, appropriate social context can facilitate losing weight in some women. One of the women said, *“If I want to lose weight, it’s because of what others say. They encourage me to do that.”*

### 3.2 Theme 2. Being Different

Feeling of being different was a basic experience of living with obesity in women. This feeling was mentioned by one of the participants before and after obesity, *“When I wasn’t fat, I played with the kids, ran and was happy, but now I hate exercising.”*

Being different was not always a negative experience by women, and some of them expressed it positively. In this regard, one of them said, *“I think the fat people are more well-tempered, more kind and are better”*.

Women also found themselves physically different. Some believed they were slower than others. The experience of one of the women in doing the household was as following, *“The one thinner can quickly go and do what she wants, but I, as a fat woman, spend half an hour to move myself”*.

In return, other women considered themselves more capable than other women, *“Fat people are more resistant and less frequently get sick”*. Another participant described being different at workplace as following, *“It’s difficult for me to work in a place where there are men. They don’t treat me the same ways as they treat thin women”*.

### 3.3 Theme 3. Being Seen

Because gaining weight is a visible condition, understanding of being seen is important and disturbing for women. Most of the participants had this feeling when they ate, drank, shopped and went out. In this regard, one of them said, *“It’s as if obesity is a light grey which attracts all the attention among all human being and things”* or *“I don’t like to be seen because I’m seen badly”*. Another woman said, *“Because fat people aren’t like other people of the society, they are seen more, and they may be hurt by looks, words or sarcasms of other people”* or *“you can’t go to the society. When you go, they don’t like you as a normal person”*.

Some were afraid of eating in the presence of others. One of the women said, *“When something is to be eaten in a community, the fats are ashamed of eating”*. For one of the women, being seen in the family communion was also important, *“Because my overweight is more seen than others, I never like to take pictures, and I’ve never kept any of my photos”*.

Another woman said, *“Apart from its side effects on the body, obesity is physically awful, since when you walk, they all look at you in a special way”*. Another woman said, *“Since we look abnormal in the street, it’s discouraging.”*

## 4. Discussion

The aim of the present study was to understand the lived experience of excess weight in women. Although some of the women had positive lived experiences of excess weight, their overall statements presented negative lived experiences of excess weight, and most of them considered social judgment as one of the important factors in physical and mental health. Society and social context can be a supportive source for excessive weight people. Excess weight is not innately a negative condition, but the social context can negatively present and judge it which can cause individuals’ negative experiences (Ogend & Clementi, 2010). The themes and sub themes of the present study, attained from participants’ experiences, support the findings of previous studies attained from excessive weight people of other societies (Puhl & Hever, 2009; Creel & Tillman, 2011). In the present study, all the participants mentioned the hurt of judgment by others verbally or non-verbally. Perhaps according to Watson, outlining the importance of this finding is that the ability of nursing profession is connecting emotionally with people to provide safe care, in movements, expressions, tone of voice, touching and other nursing communicative skills (2007). Evidences of the conducted studies showed that health care providers such as physicians, nurses, dietitians and psychologists exhibited judgments against excessive weight people in parallel ways (Danielsodttir, O’Brien, & Ciao, 2010).

Based on women’s expressions, most of them did not like to be judged and evaluated by others. Being seen and being different in social context were also important to them. These issues were consistent with the results of a study by Thomas in Australia (2008). Most of the women in our study said, that people’s reactions to their weight, increased their vulnerability. The results of a study in England also stated that unpleasant lived experiences of excess weight have double effect on physical, social and psychological health in excessive weight people (Brown & Meclimens, 2012). The participants’ understanding of the hurt of judgment theme was differently emerged and expressed, and they talked about how social judgment affected their understanding and experiencing. To address these issues, most of them were aware of enjoying being different and of liking to be normal as well as considering excess weight as a taboo-induced size. They were also aware of the role of family members, friends and colleagues and their effects which made the understanding of their weight be different. Siero and colleagues in UK have shown that supporting excessive weight women by family, friends and society is among important factors in their losing weight (2014). In the present study, most of the women were dissatisfied with their physical appearance compared with others. According to National Health Research Institute (2011) in America, different appearance of excessive weight people compared with other people were painful and worried them. Also, most of the women complained about people’s and health care providers’ stunning look so that this experience led to the emergence of problems for them (Randall-Arell & Utley, 2014).

Most of the women talked about discrimination against them in the society. In a study conducted by Vartanian and Smith in Australia, the results showed that according to some excessive weight women, people’s looks were associated with stigma (2013). The findings of the present study showed that all women were harassed by the theme of the hurt of judgment and prejudice in different social contexts (family, workplace, university and health centers). The results of a study in Australia also support the above findings in that the excessive weight people are negatively evaluated in the educational setting and even in the media (Vartanian, 2011; Puhl, Luedicke & Heuer, 2011; MacCann & Roberts, 2013). The important thing is that a negative judgment against excessive weight people can make them isolated, and encouragement can motivated them to participate in society. For instance, Puhl and colleagues conducted a study on a group of excessive weight people in America to present society’s attitude towards them. The results showed that addressing them by words such as obese and overweight led to highest stigma for them. Instead, calling them by words such as unhealthy encouraged them to have programs for losing weight (2012). In another study, the results showed that judging against excess weight in society can decrease offering necessary information to the excessive weight people (O’Brien, Puhl, Latner, Mir & Hunter, 2010). Lawrence and colleagues reported that judgment against to excessive weight people leaded to unpleasant consequences on social and emotional health and on lack of educational achievements (2010).

According to the most of the women, judgment against to them, not only had no effect on their willingness and motivation to lose weight but also made them discouraged and brought them mental and emotional stress. The findings of the studies showed that judging against the excessive weight people decreased their motivation to change diet and exercise behavior to lose weight which subsequently accelerates excess weight -induced problems (Vartanian & Smith, 2013; King & Puhl, 2014; Kleinman & Hall, 2006). This agrees with the finding of a study by Wanat and colleagues, that social judgment against to excessive weight people has always a great impact on health and life quality (Wanat et al., 2014).

In the present study, the findings represented that negative attitude towards the excessive weight women threaten their health and impose mental and emotional stress or even physical problems on them. In this regard, the results of a study in England showed that the weight stigma was not an appropriate tool for the excessive weight subjects to lose weight. Instead, it can function as an obstacle or disorder against efficient efforts in excess weight interferences (Puhl, Peterson, & Leudicke, 2012; Latner & Ciao, 2013). In this study, stereotypes of prejudice and discrimination against the excessive weight women are seen in multiple dimensions of life and are repeatedly expressed by the participants. In this regard in a review study, the results showed that the viewpoint of physicians towards the excessive weight people affected their treatment so that physicians felt that treatment was not effective on the excessive weight people (Bomback, 2014). Most of the women in the present study talked about mixed feelings such as being reluctant, anxious and worried about their weight when they received health services. The results of various studies showed that psychological and social problems related to excess weight and discrimination in receiving health services were very important to the women, and their negative consequences made them to be reluctant about health warnings and consider them insignificant (Merrill & Grassley, 2008; Winsome Beverly, 2011). In this regard, the results of a phenomenological study showed that excessive weight women postponed screening tests such as Pop Smears and breast cancer. They were also afraid of confronting with health care providers to receive health services (Spenser, 2011). The hurt of judgment was as a strong felling in all women due to their weight and created anxiety during their interaction. In this regard, the results of a study in Australia revealed that many excessive weight people believed their weight was the first highlighted sign when confronted with health care providers (Varatanian & Smith, 2013). In the present study, considerable concern was evident in the physical appearance of women so that most of them were dissatisfied with some parts of their bodies such as the abdomen, breasts and arms. In this regard, Gavin and colleagues asserted that women perceived weight in different ways. Understanding excessive weight women’s about themselves can cause chronic psychological stress which is a risk factor for diabetes, hypertension, high cholesterol and heart disease (2010).

Generally speaking, most of the participants in the present study experienced discrimination and stigma in the society. The documents showed that psychological factors such as discrimination can play a vital role not only in mental health but also in physical health. In the present study, although social interactions were often difficult for participants, a few of them talked positively about communicating with other people and enjoyed being in touch with other people.

## 5. Conclusion

The present study aimed to explore the meaning of lived experience of excess weight in women, and it portrayed just a small portion of excessive weight in Iranian women’s lives. The study revealed that Iranian excessive weight women found the hurt of judgment is a important factor of living with excess weight, and factors such as social judgment, being different and being seen are important to them. These findings can prove helpful in promoting the nursing knowledge concerning the process of communicating to increasing population. The achievement of the present study was the fact that if the excessive weight women experience the hurt of judgment due to their weight in the society, especially by families and health care providers, its negative outcomes make them not receive health services or participate in the society and lack of effective communication in the family. All the above factors have negative impacts on women’s health and consequently on the families and society. In other words, professional and scientific attitude of nurses and health care providers towards excess weight phenomenon promotes accessibility and sustaining trust in excessive weight patients and also promotes the possible lookup and access to health care and receiving high quality services. Health care providers have to gain more knowledge about the interaction with excessive weight people and to care of them based on their experiences.

In summary, this study revealed the experiences of women’s living with excess weight. The purpose of the study was to expose a deep understanding about how women’s living with excess weight was felt and expressed.
